# Investigating attachment, caregiving, and mental health: a model of maternal-fetal relationships

**DOI:** 10.1186/s12884-014-0383-1

**Published:** 2014-11-19

**Authors:** Judi Walsh, Erica G Hepper, Benjamin J Marshall

**Affiliations:** School of Psychology, University of East Anglia, Lawrence Stenhouse Building, Norwich Research Park, Norwich, UK; School of Psychology, University of Surrey, Guilford, UK

**Keywords:** Antenatal attachment, Caregiving, Mental health, Pregnancy, Relationships

## Abstract

**Background:**

Maternal-fetal relationships have been associated with psychosocial outcomes for women and children, but there has been a lack of conceptual clarity about the nature of the maternal relationship with the unborn child, and inconsistent findings assessing its predictors. We proposed and tested a model whereby maternal-fetal relationship quality was predicted by factors relating to the quality of the couple relationship and psychological health. We hypothesized that the contribution of individual differences in romantic attachment shown in past research would be mediated by romantic caregiving responsiveness, as maternal-fetal relationships reflect the beginnings of the caregiving system.

**Methods:**

258 women in pregnancy (13, 23, and 33-weeks gestation) completed online measures of attachment to partner, caregiving responsiveness to partner, mental health, and thoughts about their unborn baby. Structural equation modeling was used to test a model of maternal-fetal relationships.

**Results:**

Maternal-fetal relationship quality was higher for women at 23-weeks than 13-weeks gestation. Women in first pregnancies had higher self-reported scores of psychological functioning and quality of maternal-fetal relationships than women in subsequent pregnancies. Structural equation models indicated that the quality of the maternal-fetal relationship was best predicted by romantic caregiving responsiveness to partner and women’s own psychological health, and that the association between adult romantic attachment avoidance and maternal-fetal relationships was fully mediated by caregiving responsiveness to partner, even after controlling for other factors. These data support the hypothesis that maternal-fetal relationships better reflect the operation of the caregiving system than the care-seeking (i.e., attachment) system.

**Conclusions:**

Models of maternal-fetal relationships and interventions with couples should consider the role of caregiving styles of mothers to partners and the relationship between expectant parents alongside other known predictors, particularly psychological health.

## Background

The focus of this paper is on the relationship a woman develops with her unborn child during pregnancy (herewith referred to as maternal-fetal relationships, or MFR). Individual differences in the way mothers conceptualize their relationship with their unborn child have been associated with important outcomes including mental health, well-being, and health practices in pregnancy [1], postpartum parent-infant interaction [2], infant mood [3], and child disorganized attachment, itself a risk factor for psychopathology and poor socio-economic outcome [4,5]. Thus, understanding MFR appears vital in understanding adaptation in pregnancy and beyond [6,7], and identifying difficulties with MFR could guide risk-assessment and intervention [1]. But efforts to augment the maternal-fetal relationship have not always been successful [8-10] and some urge caution in trying to “interfere” in the maternal-fetal relationship because we know little about its nature and development [1]. The present study attempts to clarify the nature and predictors of MFR.

Research has found MFR to be associated with security, support, and satisfaction in parental and partner relationships, implying that representations of other relationships are important in MFR [11-15]. For example, Mikulincer and Florian [16] found that romantic attachment security in adult relationships was associated with quality of MFR and mental health throughout pregnancy. Some studies, though, have found no link between couple or family relationships and MFR [17,18], and even when associations are found, the underlying mechanisms remain unclear. We suggest that these inconsistencies reflect two core methodological and theoretical issues, which we address in the present study.

First, few MFR studies have used dimensional romantic attachment measures. Some earlier research [16] used attachment-style categories (secure, avoidant, anxious-ambivalent), but it is now accepted that individual differences in adult romantic attachment are best captured along two continuous dimensions of attachment avoidance and attachment anxiety [19,20]. Those high on attachment *avoidance* are concerned with independence, uncomfortable with intimacy, and deactivate the attachment system. Those high on attachment *anxiety* are preoccupied with relationships, concerned about rejection, and hyperactivate the attachment system [20]. Studies have found that attachment avoidance, but not always attachment anxiety, negatively relates to desire to have children before, during, and after pregnancy [21-23]. Therefore, the two dimensions of romantic attachment may relate to MFR via different mechanisms, which have been obscured in studies using attachment categories or general relationship security or positivity concepts.

Second, we propose that MFR, though often conceptualized as, and referred to as antenatal ‘attachment’ [24], is not indicative of the attachment system. A lack of conceptual clarity — attested by widely-varying definitions — can inhibit understanding of maternal-fetal relationships [25-27]. Various theoretical definitions and associated measures of the parental-fetal bond have emphasized different elements. For example, Cranley’s Maternal-Fetal Attachment Scale [24] measures engagement in behaviors of affiliation and interaction with the fetus. Condon’s Maternal Antenatal Attachment Scale [28] considers love for the fetus as the central experience and includes maternal dispositions towards the fetus of knowing, being with, protecting, gratifying needs, and avoiding loss [28]. Overarchingly, protection appears to be a key component in many definitions [26,29-31].

Most of the work looking at the emotional tie between parents and their unborn children is rooted in attachment theory [32], but this theory states that the attachment system involves *seeking* care from someone who can provide comfort and protection [32,33], whereas the caregiving system involves attending to need and *providing* protection [32,34]. Arguably, the mother’s antenatal relationship with her child is not attachment — parents do not (usually) seek care from their unborn children — but may be more akin to the beginnings of caregiving representations [29,30,35]. In an adult relationship, attachment security, especially low avoidance, facilitates responsive caregiving in the couple domain [36-38], and also the parenting domain [39,40]. We argue that this same caregiving system may also inform the earliest representation of a mother’s relationship with the child.

We aim to examine predictors of MFR, testing the hypothesis that individual differences in romantic attachment *indirectly* predict MFR via (i.e., are mediated by) the caregiving system. We use attachment and caregiving variables that are both assessed at the romantic level, allowing us to test which element of a couple’s relationship impacts most on maternal relationships with the unborn child. We also investigate and control for the influence of other factors shown to be linked with MFR in previous research: pregnancy-specific anxiety [41-43], mental health [17], gestational age [27] and parity [17]. In so doing, we intend to provide a more comprehensive account of the interpersonal and intrapersonal antecedents and correlates of MFR.

## Methods

### Participants and procedure

Participants were pregnant women recruited through the website BabyCentre.co.uk, an online provider of reproductive information and support. We included an online survey link in two weekly BabyCentre emails to all women registered at 13, 23, and 33-weeks gestation. Because of our research questions, only women in a relationship were invited to take part. Ethical approval was given by the University of East Anglia School of Psychology Research Ethics Committee, and therefore this study was performed in accordance with the ethical standards laid down in the 1964 Declaration of Helsinki and its later amendments. All participants gave their informed consent prior to inclusion in the study. After 408 women began the survey, 263 completed it and clicked an exit-check to submit their data. We excluded three participants not in a relationship, and two with notable missing data.

The final sample size was 258 (aged 19–45 years, *M* = 31.78, *SD* = 4.51), including 57 women at 13-weeks gestation, 94 at 23-weeks, and 107 at 33-weeks. Half (48%) were in their first pregnancy. Participants had been in their current relationship for 1.25-20.83 years (*M* = 7.12, *SD* = 3.93), and 255 reported that their current partner was the unborn baby’s father (1 was not, 2 undisclosed). This was a predominantly white, well-educated sample (89% Caucasian, 41% had a first degree).

### Measures

Participants reported demographic information and completed measures of MFR (our main outcome measure), pregnancy anxiety, couple relationship measures (romantic attachment and romantic caregiving to partner), and mental health. The order of the MFR and couple relationship measures was counterbalanced.

#### Maternal-fetal relationship

Because we were interested in a global conceptualization of MFR, and different measures of MFR may capture slightly different constructs [29], we used three scales from two different MFR measures to represent a global latent variable. The 2004 version [44] of the Maternal-Fetal Attachment Scale (MFAS [24]) includes 17 items (e.g., *I try to picture what the baby will look like*) (1 = *strongly disagree*, 4 = *strongly agree*; α = .77). The Maternal Antenatal Attachment Scale (MAAS [28]), includes 19 statements regarding the past two weeks, each rated on an appropriate 5-point scale (e.g., *totally-not at all*). It yields two scales: attachment quality (e.g., *I have felt the baby inside me is dependent on me for its well-being*; α = .78) and attachment intensity (e.g., *I have found myself talking to the baby* α = .71), which are often combined to provide a total score.

#### Romantic attachment

The Experiences in Close Relationships Scale Short-Form (ECR-S [45]) is a 12-item scale derived from the original 36-item ECR [19] (1 = *disagree strongly*, 7 = *agree strongly*). Six items assess attachment anxiety (e.g., *I need a lot of reassurance that I am loved by my partner*; α = .71) and 6 assess attachment avoidance (e.g., *I try to avoid getting too close to my partner*; α = .80). Validity is equivalent to the original ECR [19].

#### Romantic caregiving responsiveness

The Caregiving Questionnaire (CQ [37]) has 32 items each scored 1 (*strongly disagree*) to 6 (*strongly agree*). It comprises four 8 item dimensions: *proximity* (willingness to provide partner with supportive physical closeness, e.g., *When my partner is troubled or upset, I move closer to provide support and comfort*; α = .86); *sensitivity* (ability to detect partner’s non-verbal cues, e.g., *I am very attentive to my partner’s nonverbal signs for help and support*; α = .86); *cooperation* (capacity to assist without controlling, e.g., *I am always supportive of my partner’s own efforts to solve his/her problems*; α = .86); and *compulsive caregiving* (tendency to become over-involved with partner’s difficulties, e.g., *I frequently get too “wrapped up” in my partner’s problems and needs*; α = .68). Following standard practice [46], we combined the three positive scales into an overall “responsive caregiving” index (α = .89). This measure shows good 1-month test-retest reliability and good partner corroboration [37].

#### Pregnancy anxiety

The Pregnancy Anxiety Questionnaire (PAQ [42]) comprises 10 items (e.g., *I have a lot of fear regarding the health of my baby*) (1 = *not at all*, 4 = *very much*; α = .84).

#### Mental health

The 5-item Mental Health Inventory (MHI-5) is part of the Medical Outcomes Study (SF-36 [47]). It is widely used, with good internal consistency in British populations [48]. Questions refer to the past month (e.g., *How often have you felt down-hearted and blue*) (1 = *all of the time*, 6 = *none of the time*), and high scores indicate positive mental health (α = .85).

### Analysis strategy

Descriptive statistics and raw correlations are presented in Tables 1 and 2. In line with past research, romantic attachment anxiety and avoidance correlated negatively with romantic caregiving responsiveness and MFR indices, which were related. Romantic attachment anxiety also correlated with romantic compulsive caregiving, but compulsive caregiving was unrelated to MFR. Therefore, we omitted it from our main analyses. As expected, mental health and pregnancy anxiety correlated with romantic relationship and MFR indices, highlighting their relevance.

**Table 1:** **Descriptive statistics**

**Variable**	**Mean**	**SD**
1. Attachment anxiety	3.13	1.09
2. Attachment avoidance	1.95	0.98
3. Responsive caregiving to partner	4.65	0.63
4. Compulsive caregiving to partner	3.33	0.72
5. Mental health	4.79	0.65
6. Pregnancy anxiety	2.25	0.57
7. Maternal-fetal relationship: MFAS	4.11	0.47
8. Maternal-fetal relationship: MAAS quality	4.45	0.40
9. Maternal-fetal relationship: MAAS intensity	3.47	0.53

**Table 2:** **Zero-order correlations**

**Variable**	**1**	**2**	**3**	**4**	**5**	**6**	**7**	**8**
1. Attachment anxiety	–							
2. Attachment avoidance	.44	–						
3. Responsive caregiving to partner	-.33	-.58	–					
4. Compulsive caregiving to partner	.35	.01	-.20	–				
5. Mental health	-.50	-.40	.34	-.31	–			
6. Pregnancy anxiety	.30	.15	-.22	.28	-.38	–		
7. MFR: MFAS	-.18	-.30	.33	-.05	.31	-.16	–	
8. MFR: MAAS quality	-.31	-.36	.40	-.09	.37	-.25	.66	–
9. MFR: MAAS intensity	-.07	-.28	.27	.02	.13	.04	.61	.51

*Note*. Correlations > |.12| are significant at *p* < .05; those > |.16| are significant at *p* < .01.

We conducted analyses in AMOS 17.0, using partially-latent structural equation models [49]. This method provides several advantages, including ability to test model fit, compare coefficients across multiple groups, and produce the most parsimonious account of the data based on theoretical and empirical reasoning. The three measures of MFR (MFAS, MAAS quality, MAAS intensity) were modeled as indicators of a latent variable. The remaining constructs, assessed using single measures, were modeled as observed variables. To reduce impact of outliers and minor skew/kurtosis, we square-root-transformed MFR, mental health, and romantic attachment avoidance scales. No missing data were present on the composite variables.

We examined two sets of SEM. The primary model examined which of the relevant variables (i.e., romantic attachment, responsive caregiving to partner, mental health, pregnancy anxiety) best predicted MFR, and tested the hypothesized mediating role of romantic caregiving. Then, multiple-group models tested whether the observed patterns held across levels of gestational stage (13, 23, or 33-weeks) and parity (first or subsequent pregnancy). Throughout, given the correlational data, the phrase “predict” is statistical and may not reflect causality.

We evaluated model fit using a range of recommended indices [50]. These were *χ*^2^ (which tests the null hypothesis that the model does not differ significantly from the data, but is highly sensitive to sample-size so rarely nonsignificant [51]; normed-*χ*^2^ (i.e., *χ*^2^ divided by *df* to reduce influence of sample-size: good if ≤2 [52]; comparative fit index (CFI: good if ≥ .95); root-mean-square error approximation (RMSEA: good if ≤ .06), and standardized root-mean-square residual (SRMR; good if ≤ .08). We tested indirect (i.e., mediating) paths by running 1000 bootstrap resamples and calculating bias-corrected estimates and confidence intervals (CIs). When *comparing* model fit, we used Δ*χ*^2^ and a critical value of ΔCFI = .010 compared to the reference model [53,54]. When examining *specific* parameter estimates, we accounted for multiple tests by adopting a conservative alpha level of .01.

## Results

### Measurement model

We first tested the adequacy of the measurement model, allowing all variables to covary freely and examining factor loadings onto the MFR latent variable. The model fit reasonably, meeting two criteria, *χ*^2^(10) = 410.35, normed-*χ*^2^ = 4.04, CFI = .955, RMSEA = .109 (90% CI = .075, .145), SRMR = .052. All three MFR scales loaded strongly onto the latent variable, βs > .67, *p*s < .001.

### Primary model

**Figure 1:**
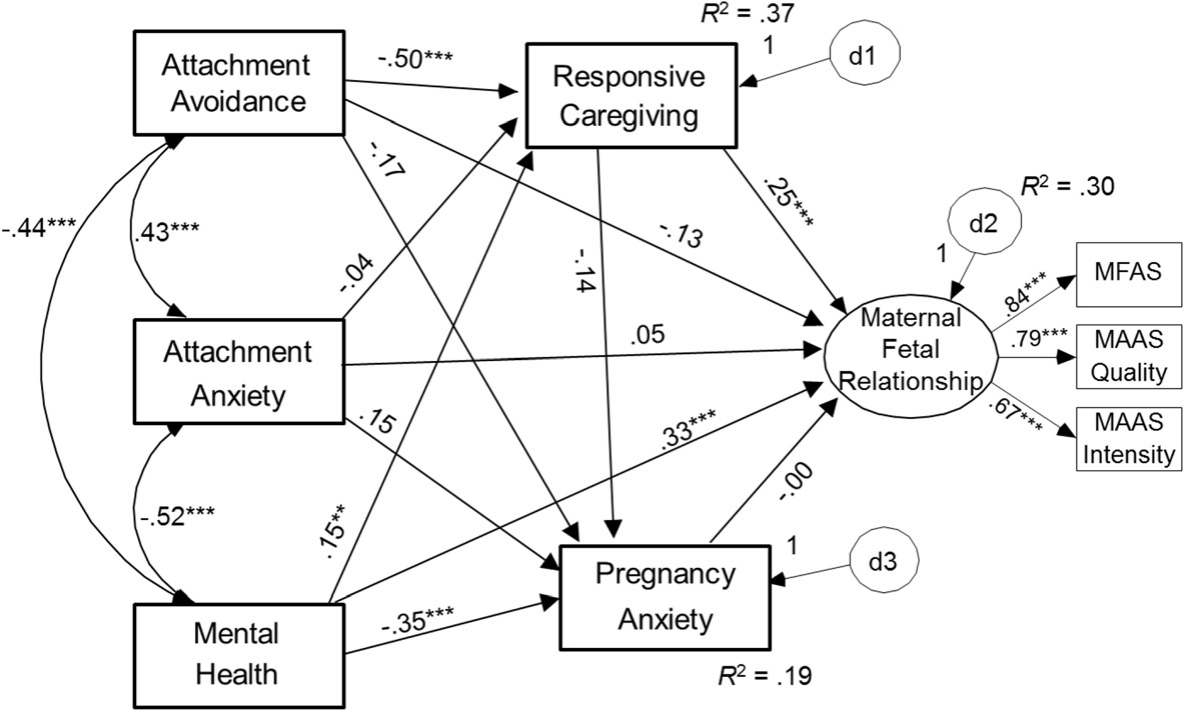
**Saturated model depicting predictors of maternal-fetal relationships.**

We tested a saturated model, examining the hypothesized predictors of MFR (Figure 1). Exogenous predictors (romantic attachment anxiety, romantic attachment avoidance, mental health) were allowed to correlate, and all pathways were included. The pathways were arranged in our hypothesized configuration, with MFR as our dependent variable. This saturated model naturally fit identically to the measurement model.

Consistent with raw correlations, mental health was associated negatively with romantic attachment anxiety, romantic attachment avoidance, and pregnancy anxiety, and positively with responsive caregiving to partner. Controlling for mental health, the paths from romantic attachment anxiety and avoidance to pregnancy anxiety (positive in raw correlations; Table 2) did not reach significance. This suggests that insecurely-attached women are anxious about pregnancy because of their generally poorer mental health. In contrast to past research, after controlling for caregiving responsiveness to partner, MFR was not significantly predicted by pregnancy anxiety or romantic attachment. Its only significant predictors were caregiving responsiveness to partner and mental health.

We next trimmed the model based on *a priori* theoretical reasoning [49], removing the non-hypothesized path between responsive caregiving to partner and pregnancy anxiety, and those between romantic attachment and MFR, which we expected to be mediated by caregiving responsiveness to partner. Removing these paths did not reduce model fit, Δ*χ*^2^(3) = 6.28, *p* = .10, ΔCFI = .005.

We further trimmed the model on empirical grounds, removing paths that were non-significant and also theoretically sensible, to achieve a parsimonious yet interpretable model. That is, we removed (a) the path from romantic attachment anxiety to romantic caregiving given that avoidance, but not anxiety, tends to relate to caregiving sensitivity or proximity [38,40,55], (b) the path from romantic attachment avoidance to pregnancy anxiety as this contradicts avoidant individuals’ deactivating strategies and reliance on distancing coping [16], and (c) the path from pregnancy anxiety to MFR given its near-zero value when controlling for other factors, plausibly implying that these are two separate facets of functioning in pregnancy. This left only the non-significant path from romantic attachment anxiety to pregnancy anxiety, which we retained given its theoretical plausibility and near-significance, and to avoid the risk of oversimplifying based on chance variation [49]. Removing these three paths did not reduce model fit, Δ*χ*^2^(3) = 2.81, *p* = .42, ΔCFI < .001.

**Figure 2:**
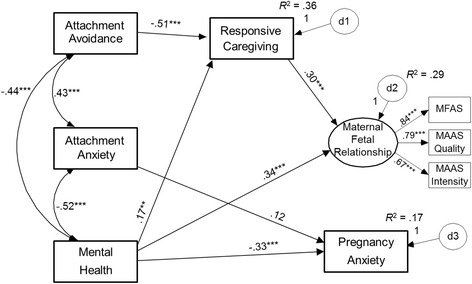
**Final trimmed model depicting significant predictors of pregnancy anxiety and maternal-fetal relationships.**

The resulting model fit remained reasonable, and all paths remained significant (Figure 2). Furthermore, as expected, significant indirect effects showed that responsive caregiving to partner mediated the associations between romantic attachment avoidance and MFR, B = −.07, SE = .02, 99% CI = [−.12, −.03], and between mental health and MFR, B = .03, SE = .01, 99% CI = [.004, .08]. This supports the argument that MFR largely reflects the operation of the caregiving system (and not the attachment system), as well as reflecting overall psychological functioning. The capacity for responsive caregiving fully accounted for the positive MFR reported by securely-attached (i.e., low-avoidant) women^a^.

### Supplementary models

#### Gestational age

We used multiple-group SEM to examine whether the patterns above (cf. Figure 2) held across the three gestational ages (13, 23, and 33-weeks). We did so in a structured means model (estimating latent means while constraining measurement intercepts equal across groups [53]) to control for the effects of gestational age on MFR. The initial model fit the data well, meeting three criteria, *χ*^2^(52) = 82.58, normed-*χ*^2^ = 1.59, CFI = .954, RMSEA = .048, SRMR = .107. We then tested a series of models constraining parameters to be equal across groups, and compared the fit of each one to this reference model. If a constraint does not worsen model fit, we can conclude that the relevant parameter(s) do not differ significantly across gestational age groups.

First, we established equivalence of factor loadings for MFR (indicating relevance of each subscale to the latent variable), which did not reduce fit, Δ*χ*^2^(4) = 7.16, *p* = .13, ΔCFI = .005. Second, we constrained the structural elements hypothesized to be equal across groups (i.e., covariances, paths, and means of attachment, mental health, caregiving, and pregnancy anxiety); model fit was again not reduced, Δ*χ*^2^(28) = 31.53, *p* = .29, ΔCFI = .005. Finally, as MFR levels might vary by gestational age, we examined their equivalence separately. Indeed, constraining MFR intercepts to be equal reduced model fit, Δ*χ*^2^(2) = 12.07, *p* = .002, ΔCFI = .016, so we allowed these to vary. The final model fit acceptably, *χ*^2^(84) = 121.10, normed-*χ*^2^ = 1.44, CFI = .944, RMSEA = .042, SRMR = .129.

The pattern of estimates was virtually unchanged from the primary model above, with all paths remaining significant or non-significant. Crucially, controlling for gestational age, romantic attachment avoidance predicted responsive caregiving to partner (β = −.51, *p* < .001), which predicted MFR (β = .37, *p* < .001), and the indirect effect remained significant, B = −.07, SE = .02, 99% CI = [−.13, −.03]. Similarly, mental health predicted responsive caregiving to partner (β = .17, *p* = .002) and also evidenced a significant indirect effect to MFR, B = .03, SE = .01, 99% CI = [.01, .08]. The model estimated the latent intercepts at 13-weeks and 33-weeks compared to 23-weeks as the reference group. MFR in women at 23-weeks gestation was significantly higher than those at 13-weeks, *Z* = 3.16, *p* = .002, but not significantly different from women at 33-weeks, *Z* = 0.39, *p* = .70.

In summary, the hypothesized model applies equally well across the timeline of pregnancy, with quality of MFR lowest at 13-weeks gestation but consistently predicted best by responsive caregiving to partner and mental health.

#### Parity

We used multiple-group SEM to test whether the same model held across women in their first versus subsequent pregnancy. The initial model fit well, meeting all criteria, *χ*^2^(34) = 62.83, normed-*χ*^2^ = 1.85, CFI = .954, RMSEA = .058, SRMR = .064. Using the same strategy as above, constraining factor-loadings reduced model fit, Δ*χ*^2^(2) = 15.51, *p* < .001, ΔCFI = .021. MAAS intensity, although highly-significant in both groups, loaded slightly more strongly onto MFR for women in their first pregnancy (β = .66, *p* < .001) than a subsequent pregnancy (β = .59, *p* < .001), *Z* = 2.28, *p* < .05. Thus, we allowed this single loading to vary across groups.

Constraining the hypothesized structural elements of the model again reduced fit, Δ*χ*^2^(13) = 38.57, *p* < .001, ΔCFI = .041. Parameter comparisons (Table 3) showed that women in their first (vs. subsequent) pregnancy reported lower romantic attachment avoidance, better mental health, and evidenced a weaker correlation between these two variables, *r*_first_ = −.38, *p* < .001, *r*_subsequent_ = −.44, *p* < .001, *Z* = 2.39, *p* < .001. Women in a first pregnancy also reported higher pregnancy anxiety and higher MFR. Thus, the final model allowed one factor-loading, four means, and one covariance to differ across groups, but held all directional paths equal across groups. This fit the data reasonably, *χ*^2^(45) = 78.62, normed-*χ*^2^ = 1.75, CFI = .947, RMSEA = .054, SRMR = .073.

**Table 3:** **Variables that differ significantly according to parity in SEM**

**Variable**	**First pregnancy**		**Subsequent pregnancy**		**Difference**
	M	SD	M	SD	Z (in SEM)
Attachment avoidance	1.63	0.63	2.24	1.14	5.08***
Mental health	4.76	0.81	4.53	0.83	2.30*
Pregnancy anxiety	2.33	0.60	2.18	0.54	3.34***
Maternal-fetal relationship (latent variable)					2.06*
MFAS	4.22	0.42	4.00	0.48	
MAAS quality	4.50	0.41	4.41	0.39	
MAAS intensity	3.65	0.47	3.31	0.52	

*Note*. *p < .05, ***p < .001. Means and SDs are provided from the raw data, whereas difference tests were based on estimated means/intercepts in SEM (using transformed variables where necessary and controlling for any predictors).

All direct and indirect paths from the primary model remained significant or non-significant accordingly. Again, the key findings remained robust: romantic attachment avoidance and mental health predicted responsive caregiving to partner, which predicted MFR, and the indirect effects remained significant (estimates and CIs identical to the gestational-age model). Thus, the hypothesized model applies equally well to women in a first or subsequent pregnancy: although they experience differing levels of wellbeing, caregiving responsiveness to partner is still a robust predictor of MFR.

## Discussion

Previous research has suggested that secure and positive adult romantic relationships are associated with quality of maternal-fetal relationships, but findings have been inconsistent, and the underlying mechanisms have not been clear. We proposed that responsive caregiving is a previously untested predictor of MFR and a key mediator in its link with adult relationships. Supporting this model, we found that the association between individual differences in romantic attachment avoidance and MFR was fully mediated by caregiving responsiveness to partner. This pattern was stable when considering other variables relevant to MFR: mental health, pregnancy anxiety, gestational age, and parity. Mental health was also a significant predictor of MFR, but part of its link was also accounted for by caregiving responsiveness to partner. Thus, our data demonstrate for the first time that mothers’ MFR is robustly predicted by responsive caregiving to partner, and support the hypothesis that MFR better reflects the operation of the caregiving system than the care-seeking (i.e., attachment) system.

Extant literature suggests that attachment insecurity may cause difficulties both in parents providing care for their offspring [56] and romantic couples providing care for their partners [57]. But the constraints on effective caregiving may differ depending on the specific attachment insecurity. In particular, we found a significant indirect pathway from romantic attachment avoidance, but not attachment anxiety, to MFR via caregiving. Mikulincer and Shaver [20] propose that caregiving deficits in adults with high avoidance stem from reduced empathic-concern for others’ needs. Indeed, high avoidance is associated with deficiencies in sensitive and responsive caregiving, mediated by lack of knowledge about how to provide support, low prosocial orientation, and low relationship commitment and intimacy [36]. It would be fruitful to examine these concepts more thoroughly in the antenatal period to pinpoint directions for providing support or intervention.

We did not find pathways from romantic attachment anxiety to low caregiving responsiveness or MFR. Past findings have often reported negative links between women’s attachment anxiety and caregiving variables [36,55,58] but not always [38]. Attachment anxiety is thought to inhibit responsive caregiving because the individual focuses on their own distress and attachment needs over the needs of others [36,58], and may lead to intrusiveness or overinvolvement by “…(coloring) caregiving motives with egoistic desires for acceptance, approval and gratitude” [55] (p.44). It may be that when controlling for their general mental health, highly attachment-anxious women are no longer inhibited by such distress or needs; moreover, they have qualities which could support caregiving, such as comfort with emotional expression and intimacy [55]. Alternatively, features of pregnancy or the antenatal relationship may prevent attachment anxiety from reducing caregiving (and thus MFR). For example, pregnancy involves little overt care-seeking from the fetus, reducing the disruptive effects of another’s expressed needs or fear of rejection, especially in relatively low-risk pregnancies (as these were). Future research could further examine whether something about being pregnant may also “spill over” into caregiving representations in the couple relationship, perhaps with altered reciprocity or expectations thereof.

Past research has found that mental health, pregnancy anxiety, gestational age, and parity predict MFR. We uncovered interesting patterns when examining these variables alongside relationship variables. First, poorer mental health related to higher levels of romantic attachment insecurity and pregnancy anxiety. This supports consistent evidence linking attachment anxiety to psychological distress [20], and recent evidence that high-demand conditions — of which pregnancy could be one — also elicit distress in highly-avoidant individuals [59,60]. But the correlations between attachment insecurity and pregnancy anxiety became non-significant controlling for mental health, implying that insecure women’s pregnancy anxiety reflects their overall wellbeing and not relationship-specific anxieties. Moreover, controlling for caregiving responsiveness to partner and mental health, pregnancy anxiety did not predict MFR, indicating that worries about pregnancy do not necessarily inhibit forming a connection with one’s unborn child. Overall, the model suggests that pregnancy anxiety and MFR are two separate factors, the former best predicted by mental health, and the latter also predicted by responsive caregiving to partner, but neither best predicted by romantic attachment. Psychological wellbeing, then, is a relevant factor to consider when examining both elements of the pregnancy experience.

Our multiple-group models, consistent with past research, found that MFR was higher at 23 and 33-weeks than 13-weeks gestation, and that both MFR and pregnancy anxiety were higher among women in their first pregnancy than those in subsequent pregnancies [27,61]. Thus, first-time pregnant women may benefit from help managing their anxieties but appear to form particularly strong antenatal relationships with their child, mitigating against possible consequences. Interestingly, we also found that women in their first (vs. subsequent) pregnancy reported higher mental health and lower romantic attachment avoidance. This pattern was not hypothesized, but is consistent with evidence that the transition to parenthood can involve stressful change, impacting wellbeing and relationship satisfaction [62]. It may also be a function of our sample, in that more avoidant or depressed women may engage relatively less in such online forums in their first pregnancy (or more in subsequent pregnancies). Further research exploring relationship processes in first and subsequent pregnancies is needed. Crucially, our key findings that caregiving to partner and mental health best predict MFR were stable controlling for these additional important factors.

We are mindful of the limitations of our study, which should be addressed in future work. Our sample comprised women registered with an online support provider, and was predominantly middle-class and reasonably well-resourced, indicative of users of this service. The processes we have identified may differ for higher-risk samples, especially those who lack social support, have significant mental health problems, or are experiencing difficult pregnancies. Because of our research questions, we recruited women with partners. This may be one reason that romantic attachment avoidance and anxiety were highly correlated: other research has found higher correlations between attachment dimensions in longer-term couples than other samples [38]. Further research should recruit women without partners; although we linked MFR to responsive caregiving to partner, it should also relate to global caregiving representations (for which new measures have been developed since the present study [63]), or caregiving towards other children. To build on our self-report findings, which focused on cognitive and affective representations of relationship processes, future research may develop behavioral measures, such as emotional facial expression when talking about the baby. Our research was cross-sectional and focused on mothers, albeit in all stages of pregnancy. Future research would benefit from longitudinal and dyadic designs following couples through pregnancy and into the postnatal period to assess whether MFR really does have long term implications for parenting. Such research could also explore other possible paths to MFR, including other characteristics of intimate relationships aside from romantic attachment dimensions.

## Conclusions

Our findings support and extend the literature on MFR and transition to parenthood. Our model may go some way to clarifying our understanding of MFR and explaining past inconsistent associations with relationship variables. We suggest that MFR is driven more by individual differences in the caregiving system than the attachment system, alongside psychological functioning. It may therefore be prudent to focus antenatal education and intervention efforts on augmenting caregiving responsiveness, especially for women high in attachment avoidance. We are not claiming that maternal-fetal relationships are synonymous with responsive caregiving: their definition needs further exploration and clarification [64], but our findings provide a direction of focus for elucidating the mechanisms underlying the established associations between couple relationships and parenting [65-67]. Thinking about MFR in terms of the caregiving system may help to clarify past findings and to guide future research and interventions.

### Endnote

^a^A supplementary test of the saturated primary model including compulsive caregiving showed that compulsive caregiving was predicted by attachment anxiety, β = .32, *p* < .001, avoidance, β = −.27, *p* < .001, and mental health, β = −.28, *p* < .001, but compulsive caregiving did not predict pregnancy anxiety, β = .11, *p* = .08, or MFR, β = .11, *p* = .12. All other paths remained significant or non-significant as in the primary model, suggesting that compulsive caregiving is not a relevant mechanism in this context.
